# Promoting physical literacy in children and adolescents with special educational needs: a scoping review of school-based and community-based interventions, characteristics, and outcomes

**DOI:** 10.3389/fspor.2026.1900203

**Published:** 2026-10-23

**Authors:** Océane Cochon Drouet, Christophe Schnitzler, Lisa Lefèvre

**Affiliations:** 1 Department of physical education, Haute École Pédagogique du Canton de Vaud, Lausanne, Switzerland; 2 Department of Special Education, Haute École Pédagogique du Canton de Vaud, Lausanne, Switzerland

**Keywords:** inclusive education, physical activity, physical literacy, school-based intervention, scoping review, special educational needs

## Abstract

Physical literacy, understood as the motivation, confidence, physical competence, and knowledge to sustain lifelong physical activity, is increasingly recognized as a key developmental goal in physical education. Yet how this multidimensional construct is conceptualized and operationalized in programs designed for children and adolescents with special educational needs remains poorly understood. This scoping review systematically mapped the existing literature on physical literacy in educational and community-based physical activity contexts for this population, following the Arksey and O'Malley (2005) framework updated by Peters et al. (2020) and reported in accordance with PRISMA-ScR guidelines. Four electronic databases (ERIC, PubMed/MEDLINE, SPORTDiscus, and Taylor and Francis) were searched, yielding 22 empirical studies published between 2012 and 2025. Data were extracted using a standardized charting form. Three principal results emerged. First, explicit engagement with the physical literacy construct is marginal: only three of the 22 studies formally grounded their work within a recognized physical literacy framework, while the majority reduced physical literacy to its motor dimension, leaving affective, cognitive, and social dimensions largely unaddressed. This reflects the conceptual fragmentation highlighted in the broader literature on physical literacy interventions. Second, only four studies were conducted in truly inclusive settings where children with special educational needs interacted directly with typically developing peers. This reveals a structural disconnect between inclusive education policy and the settings in which research on physical literacy is conducted. Third, contextual factors, such as teacher professional development, classroom climate, and peer interaction structures, were associated with program effectiveness across multiple ecological levels, though most interventions addressed only one or two of these factors simultaneously, and no study intervened across all major levels at once. The analysis concludes that larger-scale, longitudinal, and multi-level studies conducted in truly inclusive school environments and evaluated using validated physical literacy tools designed for populations with special educational needs are necessary to address these gaps.

## 1 Introduction

Physical inactivity among young people is not a new problem, but its scale remains striking. Around 80% of adolescents worldwide fail to meet recommended levels of moderate-to-vigorous physical activity (1). The trajectory is set early: activity levels begin to decline in childhood and fall sharply through adolescence (2). Schools, which reach virtually every child on a daily basis, are uniquely positioned to interrupt this pattern. Evidence consistently shows that well-designed physical education, varied in content and genuinely inclusive, can increase students' activity levels. Such approaches also shape habits that last well beyond the school years (3).

Yet inclusion remains, for many students, more a promise than a reality. Children and adolescents with disabilities or special educational needs (SEN) are disproportionately inactive, falling short of physical activity guidelines at higher rates than their peers and facing greater risks of associated health conditions (4). This reflects structural failures that go beyond individual capacities, such as poorly adapted equipment, physical education cultures that privilege normative performance, and teachers who feel underprepared for the diversity in front of them (5). Addressing this inequity calls for a socio-ecological lens, one that moves beyond attributing inactivity to individual impairment and instead attends to the multiple layers of context in which students move and learn: school policies, physical environments, and the quality of interpersonal relationships (6).

For the purposes of this review, special educational needs (SEN) are not viewed as intrinsic deficits within the child, but rather as the result of an interaction between the learner's characteristics and rigid environmental barriers, a perspective aligned with the social model of disability and the work of leading scholars in the field (7, 8). A student is identified as having SEN when they require support that is additional to or different from the general educational offer to access the curriculum effectively. Disability is understood as an evolving concept resulting from the interaction between persons with impairments and attitudinal and environmental barriers that hinder their full and effective participation in society (9). Building on UNESCO's rights-based approach, inclusive education is understood as an ongoing systemic process that responds to the needs and diversity of every learner, enables all learners to participate and achieve together, and increases participation while reducing exclusion from the cultures, curricula, and communities of local schools (10, 11). In this review, SEN encompasses autism spectrum disorder (ASD), intellectual disability, developmental coordination disorder (DCD), dyspraxia, learning disorders, and physical disabilities. Chronic medical conditions (CMC) unaccompanied by an educational need are excluded, in line with the PCC eligibility criteria defined in Section 2.1.

Within this broader framework, physical literacy (PL) has emerged as one of the most promising individual-level goals for PE. PL is understood as a holistic, multidimensional construct, integrating physical competence, motivation, confidence, cognitive understanding, and social engagement, in service of lifelong physical activity (12, 13). First conceptualized by Whitehead (12, 14), PL has since been operationalized across multiple frameworks, including the IPLA definition and the evidence-informed model proposed by Cairney et al. (13), and has been the subject of a growing body of intervention research (15). These domains are theorized to interact dynamically and reinforce one another, ideally generating a virtuous cycle in which physical competence, motivation, and knowledge mutually support sustained engagement in physical activity, with daily physical activity behavior understood as a key outcome of this developmental process rather than a constitutive domain of PL itself (13, 15).

Translating this philosophy into inclusive practice is, however, far from straightforward. PL's philosophical roots are complex, drawing on monism, existentialism, and phenomenology (12). In Whitehead's foundational conception, movement is experienced as a whole, and education must address the whole person rather than only the physical body, a principle further elaborated in the comparative definitional analysis by Edwards et al. (16). This perspective is inherently universal: it applies to all learners, which of course includes those with SEN. Yet in practice, PE assessment and intervention frequently revert to narrow, ableist conceptions of motor competence that leave disabled students at the margins (17). Prior reviews have documented this gap empirically: Pushkarenko et al. (18) found that, although inclusion is a foundational tenet of PL, its applicability to individuals experiencing disability has been assumed rather than demonstrated, and that existing assumptions around disability within PL literature require critical examination. Saxena and Shikako-Thomas (19) similarly concluded that, across 17 PL intervention studies for children with disabilities, the evidence base remains weak and that explaining intervention mechanisms or deriving solid recommendations is barely possible. Genuine inclusion therefore requires something more than accommodation, it requires abandoning paradigms built around what is presumed to be “wrong” with some bodies, and moving toward flexible, relational pedagogies that seek to honor each learner's unique movement journey within shared educational spaces (17).

Yet despite the strength of these philosophical foundations, how PL is conceptualized, operationalized, and measured in programs designed for children and adolescents with SEN remains poorly understood. The philosophical case is well made; what the field lacks is a clear empirical picture, of what programs exist, what they measure, and where the gaps lie. It is unclear how far existing programs address PL in its full multidimensional depth, and whether they take place in genuinely inclusive settings where students with SEN learn and move alongside their typically developing peers. Without this picture, practitioners, educators and policymakers cannot make informed decisions about how to design effective, inclusive, PL-based programs. To this end, the present scoping review was conducted to systematically map the existing literature and answer the following research question: How is PL conceptualized and operationalized across educational and community-based physical activity contexts for children and adolescents with SEN, and what are the characteristics of the existing evidence in terms of study design, population, intervention features, PL dimensions addressed, and setting inclusiveness?

## 2 Methods

This scoping review was conducted in accordance with the methodological framework originally proposed by Arksey and O'Malley (20) and subsequently updated by Peters et al. (21). Reporting follows the PRISMA extension for Scoping Reviews (PRISMA-ScR) checklist (22).

### 2.1 Eligibility criteria (PCC framework)

The eligibility criteria were defined *a priori* using the population–concept–context (PCC) framework recommended by the Joanna Briggs Institute (JBI) for scoping reviews. This framework guided the construction of the search strategy and screening of all identified records. The criteria are summarized in Table 1.

**Table 1:** Eligibility criteria according to the PCC framework.

PCC Element	Inclusion Criteria	Exclusion Criteria
Population	Children and adolescents (aged 6–18 years) with SEN, including ASD, intellectual disability, DCD, dyspraxia, learning disorders, and physical disabilities.	Studies focusing exclusively on typically developing children or those with adult populations only.
Concept	PL and its operationalized dimensions (physical, psychological, social, and cognitive) and/or components (e.g., motor competence, physical competence, fundamental movement skills, physical self-efficacy, and movement motivation). Both explicit PL conceptualization and implicit operationalization were eligible for inclusion.	Studies that address only general health outcomes (e.g., BMI, cardiovascular fitness) without any reference to PL components or their development.
Context	Any school-based setting (including mainstream, inclusive, adapted, or specialized/segregated schools), community-based program, or structured home-based intervention aimed at promoting physical activity or physical education with an educational or developmental goal.	Strictly laboratory-based experimental studies or pure clinical rehabilitation settings with no educational or community-based physical activity promotion components.

SEN, special educational needs; ASD, autism spectrum disorder; DCD, developmental coordination disorder; PL, physical literacy.

### 2.2 Information sources and search strategy

A comprehensive and systematic literature search was conducted across four major electronic academic databases covering the fields of education, sports science, and health: ERIC (Education Resources Information Center), PubMed/MEDLINE, SPORTDiscus, and Taylor and Francis. The search was carried out by a team of six researchers: three senior researchers and three research assistants. Each of the four databases was assigned to a dedicated team member responsible for adapting and executing the Boolean search strategy to the specific syntax, controlled vocabulary, and subject headings (e.g., MeSH terms in PubMed) of that database. Searches were conducted between January and March 2026. No restrictions were placed on the publication year. Only peer-reviewed articles published in English or French were eligible for inclusion.

The search string was structured around three conceptual domains combined using the Boolean operator AND.

1. Physical Literacy Domain: “physical literacy” OR “motor competence” OR “physical competence” OR “fundamental movement skills” OR “physical self-efficacy” OR “movement motivation”
2. Inclusion & SEN Domain: “inclusion” OR “inclusive education” OR “inclusive physical education” OR “special educational needs” OR “SEN” OR “disabilit*” OR “autism spectrum disorder” OR “intellectual disabilit*” OR “learning disorder*” OR “dyspraxia” OR “developmental coordination disorder”
3. Population Domain: “child*” OR “adolescent*” OR “youth” OR “student*” OR “pupil*”
4. The search string was anchored in the most widely and consistently indexed PL-adjacent terms across the four databases. Terms specific to cognitive PL dimensions (e.g., movement knowledge, implementation intentions) and social PL dimensions (e.g., social participation through movement, cooperation) were not included as primary search terms, as these constructs are inconsistently indexed in the databases searched. This methodological choice is acknowledged as a limitation of the search strategy and is discussed further in Section 5.

### 2.3 Selection of sources of evidence

After the search strategies were executed, all identified records were exported and imported into Zotero reference management software. Duplicates were automatically removed by the software and subsequently verified manually by the research team. The screening process involved two sequential and independent stages designed to minimize the selection bias. In the first stage, title and abstract screening, two reviewers independently assessed all unique records against the predefined PCC eligibility criteria (Table 1). Articles that did not meet the criteria were excluded at this stage. Discrepancies between reviewers were resolved through discussion until consensus was reached; a third senior reviewer was available to adjudicate persistent disagreements. In the second stage, full-text assessment, the complete texts of all potentially relevant articles were retrieved and independently assessed by the same two reviewers. The primary reasons for exclusion at this stage (e.g., wrong population, segregated context without any educational or developmental goal, wrong concept) were systematically recorded and are reported in the PRISMA-ScR flowchart (Figure 1).

**Figure 1:**
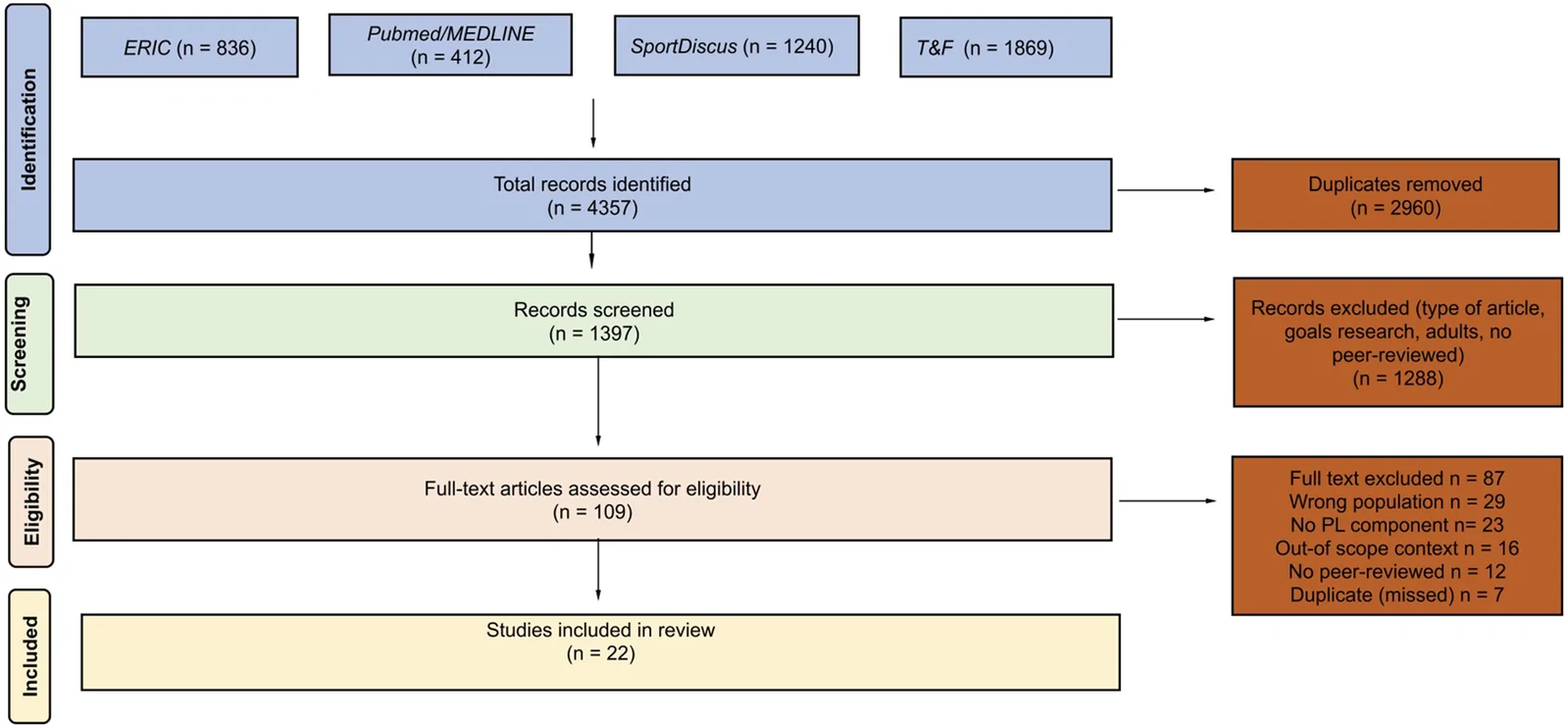
Prisma flowchart of the identification process of the scoping review.

### 2.4 Data charting process

Data from all included studies were extracted using a standardized charting form (data extraction canvas) developed specifically for this review. The charting form was first piloted independently by two researchers on a random sample of five included studies. Discrepancies in extracted data were discussed and resolved by consensus, and the form was refined accordingly before full application. Each of the 22 included articles was then assigned to one researcher for primary extraction. All completed extraction forms were subsequently cross-checked by a second senior researcher, who verified the accuracy and completeness of each entry. Disagreements at this stage were resolved through discussion, with a third senior researcher available for adjudication where consensus could not be reached.

The charting form captured the following categories:

- Study characteristics: author(s), year, country, and study design (qualitative, quantitative, mixed-methods, RCT, cross-sectional, quasi-experimental, or conceptual).
- Participant profiles: sample size, age range, and specific type of SEN.
- Context: specific setting (mainstream PE, inclusive community sports, specialized school, active recess, etc.). Settings were classified into four categories based on the degree of interaction between SEN and typically developing participants: genuinely inclusive, partially inclusive, segregated or specialized, and unclear. The settings for each article are provided at the point of reporting in Section 3.6.
- Conceptualization of PL: whether PL was explicitly defined, implicitly operationalized, or absent, and which theoretical framework was referenced. PL conceptualization was classified into three levels based on the following decision rules. Explicit was coded when at least two of the following three criteria were met: (1) PL is named in the title or stated objectives; (2) PL is measured as a primary outcome using a validated multidimensional PL tool (e.g., CAPL, CAPL-2); (3) a formal PL theoretical framework is explicitly cited [e.g., (12, 13, 23)]. Implicit was coded when PL was not named but multiple PL dimensions were addressed through intervention components or outcome measures. Absent was coded when studies targeted isolated motor or health outcomes with no reference to PL as a multidimensional construct.

Table 2 illustrates the application of these criteria to representative and ambiguous cases.

- Assessment methodologies: specific tools used (accelerometry, validated questionnaires, interviews, observation protocols).
- PL dimensions addressed: specific domains of PL (physical, affective, cognitive, social) targeted or measured.
- Ecological factors: multi-level ecological frameworks or involvement of families and communities beyond the school setting.
- Technology: use of digital tools, connected devices, mobile applications, or exergaming.
- Key findings, conclusions, and recommendations reported by the authors.

**Table 2:** Examples of application of PL conceptualization coding criteria.

Article	Criterion 1: PL named in title/objectives	Criterion 2: Validated multidimensional PL tool used	Criterion 3: Formal PL framework cited	Code	Decision
Veljkovic et al. (34)	No	No: BOT-2	No	Absent	Single dimension (motor proficiency) only; no PL reference in the study
Curtin et al. (29)	Yes	Yes: CAPL-2 + TGMD-3	Yes: Moss (2015), Cairney et al. (2019)	Explicit	All 3 criteria met
Garcia et al. (38)	No	No: Fitbit	No	Absent	PA engagement and sedentary behavior only; no PL dimension addressed
Hassani et al. (28)	Yes	Yes: CAPL	Yes: IPLA (2017), Whitehead (2013), Cairney et al. (2019)	Explicit	All 3 criteria met
Firouzjah et al. (32) *	Yes: title + objectives	Yes: CAPL-2 (primary outcome)	No: no formal PL framework cited	Explicit	Criteria 1 & 2 met; PL is the primary declared outcome operationalized through a validated multidimensional tool. Absence of Criterion 3 alone does not override the explicit PL focus of the study.
Noetel et al. (27)	No	No	No	Implicit	FMS described as “major component of PL”; multiple dimensions targeted without naming PL

CAPL, Canadian Assessment of Physical Literacy; CAPL-2, Canadian Assessment of Physical Literacy, Second Edition; TGMD-3, Test of Gross Motor Development, Third Edition; BOT-2, Bruininks-Oseretsky test of motor proficiency, second edition; FMS, fundamental movement skills; PA, physical activity. The ambiguous case * illustrates the decision rule applied when Criterion 3 is absent.

To ensure coding consistency, a random subset of 10 articles was independently double coded by two reviewers. Perfect inter-rater agreement was achieved (Cohen's *κ* = 1.00), confirming the reliability of the classification criteria. Setting classification was similarly subject to independent double-coding on the same subsample of ten articles, using the four-category operational framework defined above. Full agreement was reached on all ten cases without requiring adjudication, reflecting the clarity of the operational criteria for most studies. The two cases classified as “partially inclusive” (24, 25) were discussed and agreed upon by the research team prior to final coding.

Figure 1 shows the flow diagram for the literature selection process. This structured charting process enabled a systematic mapping of the literature across all three questions.

## 3 Results

### 3.1 Characteristics of included studies

Table 3 shows a general description of the selected studies. The 22 included studies were published between 2012 and 2025, with 18 of the 22 appearing after 2018, reflecting the recent acceleration of research interest in inclusion of SEN students. Thirteen countries are represented across the 22 articles. The United States is most frequently represented (*n* = 8), followed by Italy and Iran (*n* = 2 each). Most studies were quantitative (86.4%) and involved small samples of SEN participants (72.7% with fewer than 30 SEN participants). Most populations studied consisted of children and adolescents with ASD, intellectual disabilities, and mixed SEN profiles (31.8% each). School-based settings predominated, though three studies were conducted entirely via remote online platforms.

**Table 3:** Description of the studies retained in this scoping review.

Year of publication	N	Percent
2012–2015	4	18.20
2016–2018	2	9.10
2019–2021	5	22.70
2022–2023	2	9.10
2024–2025	9	40.90
Sample size [n SEN participants]		
<10	3	13.60
10–29	13	59.10
30–99	2	9.10
100–499	2	9.10
>500	2	9.10
SEN population		
ASD	7	31.80
Intellectual Disability	7	31.80
Mixed SEN	7	31.80
Physical Disability	1	4.50
Educational context		
Primary school	2	9.10
Secondary school	6	27.30
Mixed	5	22.70
Unspecified	9	40.90
Method		
Quantitative	19	86.40
Mixed Methods	3	13.60
PL conceptualization		
Explicit	3	13.60
Implicit	6	27.30
Absent	13	59.10
PL dimensions addressed		
Physical only	7	31.80
Physical and affective	5	22.70
Physical, affective and cognitive	3	13.60
Physical, affective, cognitive and social	2	9.10
Physical, affective and social	1	4.55
Physical and cognitive	1	4.55
Physical and social	1	4.55
Affective only	1	4.55
Affective, cognitive and social	1	4.55
Technology role		
Intervention tool	5	22.70
Measurement only	10	45.50
None	7	31.80
Setting		
School based (segregated/specialized)	8	36.40
School based (genuinely inclusive)	4	18.20
School based (partially inclusive or unclear)	4	18.20
Community-based	3	13.60
Remote/online	3	13.60

In accordance with the methodology of exploratory reviews (21), no formal quality assessment was conducted for the included studies. This decision reflects the review's mapping objective, which is to characterize the scope and nature of the evidence rather than to assess its quality. However, this choice has implications for how the results should be interpreted: the results of the single randomized controlled trial (RCT) (26) and the cluster RCT (27) carry greater inferential weight than those from quasi-experimental, pilot, or single-subject study designs, and should not be considered equivalent data. When data on effect size were available (8 out of 22 studies), they were reported to provide an indication of the order of magnitude; in their absence, directional results are described with appropriate epistemic caution.

Table 4 presents a summary of the 22 included studies.

**Table 4:** Summary of studies and distribution of physical literacy dimensions across populations, settings, and PL frameworks (*N* = 22 studies).

Study	Population	Methodological Approach	Setting	PL Frame	Physical	Affective	Cognitive	Social	Effect Size	Main Results
Bentzen and Malmquist (24)	2,651 Norwegian adolescents of 13–19 years without disabilities and 328 with mixed disabilities	Quantitative	PAR	IMP		X	X		X	Adolescents with disabilities experienced significantly lower psychological need satisfaction in physical education and organized sports compared to their typically developing peers. In contrast, self-organized physical activity showed minimal disparities between groups, suggesting it may be a more equitable context for this population
Campagna et al. (51)	19 American adolescents 12–14 years with ID, ASD, and Down syndrome	Mixed methods (qualitative and quantitative)	SEG	ABS	X	X				The six weeks school-based running program significantly improved cardiovascular fitness and enhanced self-reported quality of life. These improvements were statistically significant. Informal reports from parents and teachers also suggested positive impacts on sleep, behavior, and focus in the classroom.
Chiva-Bartoll et al. (25)	25 Spanish children mean age 10.1 years with ASD	Mixed methods (qualitative and quantitative)	UNK	IMP	X			X		The Service-Learning program significantly increased moderate physical activity levels on weekdays and improved manual dexterity and balance in children with ASD. Both parents and teachers reported social benefits, including better peer interaction and a shift toward more inclusive attitudes through the intervention.
Curtin et al. (29)	Six American adolescents 12–16 years with ID	Quantitative	UNK	EXP	X	X	X		X	This pilot intervention demonstrated high attendance and satisfaction, with positive trends in motor skills and physical activity motivation for youth with ID. The program effectively improved food literacy knowledge, particularly in categorizing food groups and identifying healthier meal options.
Firouzjah et al. (32)	30 Iranian children 8–12 years with ASD	Quantitative	SEG	EXP	X	X	X			Physical activity significantly improved total physical literacy scores and all its subscales (motivation, competence, and knowledge) in children with ASD. The intervention also enhanced cognitive performance, specifically showing significant improvements in planning, accuracy, and reaction time.
Garcia et al. (38)	42 adolescents 13–19 years with ASD (level 1)	Mixed methods (qualitative and quantitative)	SEG	ABS	X					A 12-week school-based Fitbit program proved highly feasible and well-accepted, with 76% of students with ASD achieving daily wear-time compliance. Teachers reported that students were motivated by step-tracking and friendly competition, finding the technology a useful tool for monitoring health
Giuriato et al. (39)	18 Italian children and adolescents 9–17 years with Down syndrome	Quantitative	SEG	ABS	X					The 15-week tele-coaching intervention significantly improved balance and reduced systolic blood pressure among children and adolescents with Down syndrome. While motor skills and cardiovascular health enhanced, no significant changes were observed in cognitive performance or adiposity parameters during the pilot.
Gobbi et al. (35)	19 Italian adolescents mean age 17.4 years with mild to moderate ID	Quantitative	INC	IMP	X	X			X	Peer-tutored physical education resulted in significantly higher enjoyment, perceived exertion, and light physical activity levels compared to standard PE classes. The program was particularly effective for students with intellectual disabilities who were overweight, reducing their overall inactive time.
Hassani et al. (28)	30 Iranian children 8–12 years with high-functioning ASD	Quantitative	SEG	EXP	X	X	X		X	The program significantly increased all aspects of physical literacy in children with ASD, including physical competence, daily behaviour, motivation and confidence, and knowledge and understanding. Significant improvements were observed in the experimental group from pre-test to post-test across all measured PL components, with large effect sizes reported for most outcomes.
Hawks et al. (37)	15 American children 7–16 years with severe ASD	Quantitative	SEG	ABS	X		X			The intensive camp allowed all children with ASD to acquire bicycle motor skills, with 60% achieving independent two-wheel riding. Better initial motor coordination and lower social communication deficits were identified as significant predictors of faster skill acquisition
Healy and Marchand (44)	13 American children 6–16 years with ASD	Quantitative	UNK	ABS	X	X	X	X		The Facebook-based, parent-mediated intervention was reported as a feasible and satisfactory method for supporting physical activity in children with ASD. While parents found the components effective, there was no statistically significant change in children's physical activity levels from pre- to post-test.
Jin and Yun (41)	503 South Korean middle school students (including 22 with disabilities) with various SEN	Quantitative	INC	ABS	X	X			X	Multilevel analysis revealed that teacher behavior and student implementation intentions significantly predicted physical activity levels in inclusive middle school PE. Environmental factors like lesson content and class location were found to moderate the impact of teaching behavior on student activity.
Jin et al. (40)	241 American children and adolescents 5–15 years with various disabilities	Quantitative	UNK	ABS		X				Participation in daily physical activity significantly mediated the relationship between program enjoyment and perceived general health for older adolescents in PE. This relationship was not significant for younger children in recess, highlighting the importance of structured PE for health promotion in older youth with disabilities.
Kaya and Torun (52)	20 Turkish male children 12–16 years with Down syndrome and ASD	Quantitative	SEG	ABS	X					Montessori-based education significantly improved physical fitness components, specifically speed (plate tapping) and explosive power (long jump), in youth with Down syndrome and ASD. The method supported the development of motor skills through tactile materials and freedom of movement compared to traditional programs.
Lai et al. (45)	10 American adolescents mean age 17 years with various mobility disabilities	Mixed methods (qualitative and quantitative)	SEG	ABS	X					School-based virtual reality exercise achieved high attendance and intensity, while home-based exercise without coaching failed to successfully engage participants. Supervised VR gaming at school provided a mental break from stressors and was found to increase students' energy levels and well-being
Noetel et al. (27)	238 Australian children 8–11 years with ID	Quantitative	SEG	IMP	X	X				A low-intensity blended teacher-professional learning program did not significantly improve fundamental movement skills or physical activity in children with ID. Results may have been affected by the COVID-19 pandemic and high baseline activity levels in the schools' standard programs
										
Salaun et al. (36)	23 French adolescents mean age 15.1 years with mild ID and obesity	Quantitative	SEG	ABS	X	X			X	A 9-month program successfully improved body composition, reducing waist circumference and body fat percentage in obese adolescents with ID. Participants also showed a significant decrease in body image dissatisfaction, while their global self-esteem remained stable throughout the intervention.
Shen et al. (26)	38 Chinese adolescents 15–17 years with mild to moderate ID	Quantitative	SEG	IMP	X	X	X	X	X	The program significantly improved fundamental motor skills, upper and lower limb strength, and cardiorespiratory fitness in adolescents with ID. Improvements were also found in quality of life, which showed strong positive correlations with the development of motor skills and physical fitness.
Stanish and Temple (46)	20 American adolescents 15–21 years with mild and moderate ID	Quantitative	INC	ABS	X				X	A 15-week peer-guided exercise program led to significant improvements in abdominal muscular endurance, cardiovascular fitness, and BMI among adolescents with ID. The reciprocal peer support model effectively engaged youth in structured exercise within a community-based YMCA setting.
Stanish et al. (42)	35 American adolescents with ASD (13–21 years) compared with 60 typically developing peers	Quantitative	UNK	ABS		X	X	X		Adolescents with ASD reported lower enjoyment of team sports and gym class and faced more barriers, such as fear of injury and weather, than typically developing peers. Despite these challenges, most felt physical activity was fun and good for them, and many expressed a desire to be more active.
Veljković et al. (34)	32 Serbian adolescents mean age 17.8 years with ID	Quantitative	SEG	IMP	X					The manipulative skills intervention program significantly improved the motor proficiency and body coordination of adolescents with mild intellectual disabilities in the experimental group. Significant improvements were observed in both the total motor proficiency score and the Body Coordination with *p*-values of 0.00 for both. These findings indicate a positive effect of the intervention on motor skills
Viorella et al. (43)	1 Indonesian child 12 years with mild ASD	Quantitative	INC	ABS	X	X		X		The specialized physical activity program significantly enhanced motor skills, social interaction, and positive attitudes in a student with mild autism. Improvements were specifically noted in muscle strength and the ability to recognize and respond to social and emotional cues.

SEN, special educational needs; ASD, autism spectrum disorder; ID, intellectual disability; PL Frame, physical literacy conceptualization; Setting: INC, genuinely inclusive (typically developing peers with structured interaction); SEG, segregated/specialized; PAR, partially inclusive; UNK, unclear; PL Frame: EXP, explicit (≥2 of 3 criteria met); IMP, implicit (multiple PL dimensions without PL terminology); ABS, absent (isolated motor/health outcomes); X, present; PE, physical education.

### 3.2 Conceptualization and operationalization of PL

PL conceptualization was classified into three levels: explicit, implicit, and absent, according to the operational definitions and decision rules detailed in Section 2.4. Perfect inter-rater agreement was achieved on a random subset of 10 double-coded articles (Cohen's *κ* = 1.00).

#### 3.2.1 Explicit PL

Only three of the 22 studies explicitly conceptualized PL. Specifically, Hassani et al. (28) and Curtin et al. (29) provided the most formally grounded PL frameworks. Hassani et al. (28) used the IPLA (23) definition and Whitehead's (14) phenomenological and monistic model of PL, alongside the Cairney et al. (13) PL–physical activity–health framework and self-efficacy theory (30). On their side, Curtin et al. (29) defined PL as “the ability, confidence, and desire to be physically active for life” (31), drawing on Cairney et al. (13) conceptual model and Social Cognitive Theory (30). Both studies designed their interventions explicitly around PL development and used PL as the primary measurement focus. In contrast (32), also targeted PL as their primary outcome, operationalizing it through the CAPL-2 multidimensional assessment tool and measuring all four PL subscales. However, this study did not formally cite a PL theoretical framework grounding its conceptualization at the operational rather than theoretical level.

#### 3.2.2 Implicit PL

Six other studies implicitly operationalized PL by focusing on several dimensions without using the terminology of PL. For example, Noetel et al. (27) identified fundamental skills as a “major component of PL” (33) within the context of a school-wide intervention. Similarly, Veljković et al. (34) whose manipulative skills program targeted motor control and body awareness while Shen et al. (26) addressed motor skills, physical fitness, quality of life, and social participation within an ICF-based framework. Bentzen and Malmquist (24) examined the fulfillment of psychological needs in various physical activity contexts using SDT and the family of participation-related constructs (fPRC). In addition, Chiva-Bartoll et al. (25) focused on motor skills and social inclusion through a service-learning model. Finally, Gobbi et al. (35), who measured physical activity, enjoyment, and peer-facilitated engagement in a peer-led physical education program.

Of the remaining 13 studies, none conceptualize PL holistically. Instead, they focused on isolated outcomes related to motor skills, physical fitness, or behavior, and made no reference to PL dimensions beyond the physical aspect.

#### 3.2.3 Distribution of PL dimensions

When PL dimensions were present, explicitly or implicitly, the physical dimension was addressed in 19 of the 22 studies (86%). This predominance is striking, even in studies that nominally target multiple outcomes, motor skill proficiency tends to serve as the sole proxy for PL development. The affective dimension (enjoyment, motivation, self-efficacy, and physical self-perception) was addressed in 14 studies (64%). In comparison, the cognitive dimension (movement knowledge, executive function, and implementation intentions) appeared in only eight studies (36%), and the social dimension (peer interaction, social participation, and inclusive engagement) in five studies (23%). Notably, no study outside the three explicit PL studies addressed all four dimensions simultaneously. The co-occurrence of three or more dimensions was rare in the implicit and absent categories, reflecting a fragmented and reductionist approach to PL across the SEN literature. Table 4 presents a complete summary of PL dimensions used on the 22 studies.

#### 3.2.4 Assessment tools

Assessment instruments for PL dimensions varied widely across the corpus. For the physical dimension, the Test of Gross Motor Development (TGMD-2 and TGMD-3) was used in four studies and the Bruininks-Oseretsky Test of Motor Proficiency (BOT-2) in two, supplemented by standardized fitness batteries including the Eurofit (*n* = 2), the 6-Minute Walk Test (*n* = 3), and force and flexibility tests. Affective dimensions were typically captured through self- or proxy-reported questionnaires, most commonly the Physical Activity Enjoyment Scale (PACES; *n* = 2) and the Motivation and Confidence subscale of the CAPL-2 (*n* = 2). Only two studies, Hassani et al. (28) and Firouzjah et al. (32) employed a comprehensive multidimensional PL assessment tool (CAPL and CAPL-2 respectively) designed to capture physical, affective, cognitive, and behavioral dimensions simultaneously. The PSI-VSF-ID, an instrument specifically adapted for adolescents with intellectual disability, was used in two studies to assess physical self-concept (29, 36). No study reported validating a PL-specific instrument on an SEN population as part of its methodology. The absence of multidomain PL assessment tools adapted and validated for SEN populations constitutes a significant methodological gap in the field.

### 3.3 Interventions and their effects on PL dimensions

Across the 22 included studies, interventions varied considerably in design, duration, and intensity. Programs ranged from a single-week intensive camp (37) to a nine-month longitudinal adapted physical activity program (36). Most interventional studies reported positive directional effects on at least one PL dimension (*n* = 16). Four reported mixed effects, one reported null effect (27). The last one reported only implementation outcomes, without efficacy data (38). Effect sizes were reported in only eight of the 22 studies, limiting cross-study comparison.

#### 3.3.1 Physical dimension

The physical dimension appeared in 20 studies. The data show that higher frequency, longer duration, and greater task specificity produced larger and more reliable motor gains. More precisely, Shen et al. (26), reported large effects in a six-month RCT with adolescents with intellectual disabilities, across fundamental motor skills (TGMD total: *η*² = 0.330), upper and lower limb strength, and cardiorespiratory fitness. Veljković et al. (34) obtained comparable results over 12 weeks with a manipulative skills program (BOT-2: *F* = 48.65, *p* = 0.000). Short, intensive programs also yielded measurable gains when task specificity was high: the one-week iCan Bike camp (37) produced motor skill acquisition in all 15 participants with ASD, with nine achieving independent cycling. By contrast, Noetel's et al. (27) study found no significant effects. Indeed, despite the largest sample in the corpus (*N* = 238, cluster RCT across 20 schools), this study found no significant group-by-time effects on FMS (*b* = 1.07, 95%CI [−3.70, 5.84], *p* = 0.658). The program involved low-intensity teacher professional learning rather than direct motor instruction, and teacher completion rates were the only significant predictor of student outcomes (*b* = 10.40, *p* = 0.016). This result, read alongside the positive findings from Shen et al. (26) and Veljković et al. (34), points to a distinction that is consequential for practice: it is not the existence of a school-based program that drives motor gains, but the intensity and directness of the physical instruction it delivers. Tele-coaching adds a further nuance. Giuriato et al. (39) showed that a 15-week remote program improved balance in adolescents with Down syndrome (*p* = 0.002) and systolic blood pressure (*p* = 0.04), but overall motor coordination and cardiorespiratory fitness did not reach significance.

#### 3.3.2 Affective dimension

14 studies addressed affective outcomes. Two patterns stand out. First, programs that explicitly target motivational and affective constructs, rather than treating enjoyment as a secondary outcome, report substantially larger effects. For example, Hassani et al. (28) obtained large effect sizes for motivation and confidence (*d* = 3.96) and total PL (*d* = 3.94) using a program grounded in fundamental skills play and visual communication support for children with ASD. Salaun et al. (36) demonstrated that a 30-week affectively oriented APA program significantly reduced body image dissatisfaction (*Δ* = −1.83, *p* ≤ 0.01) in obese adolescents with intellectual disabilities, with positive illusory bias emerging as a moderating variable. Gobbi et al. (35) showed that peer-tutored PE produced higher enjoyment than standard PE (PACES: *η*² = 0.276), with the strongest gains among students who were overweight.

Second, the longitudinal and observational data show a more worrying picture. Bentzen and Malmquist (24), with three years of data from 328 adolescents with disabilities, found consistently lower psychological need satisfaction in PE and organized sport compared to typically developing peers (Autonomy: *d* = 0.29; Competence: *d* = 0.30), with no significant differences in self-organized physical activity. Jin et al. (40) add a contextual qualifier: enjoyment mediated the relationship between school-based PA and general health only for older children (12–15 years) in structured PE, not for younger children in unstructured recess, indicating that the motivational function of enjoyment is context- and age-dependent.

#### 3.3.3 Cognitive dimension

Eight studies addressed cognitive outcomes. Three studies measured executive functions or movement knowledge as primary outcomes. Firouzjah et al. (32) showed that a 24-session program improved planning, accuracy, and reaction time in children with ASD (all *p* < 0.05), alongside gains on all CAPL-2 subscales, one of the few studies where motor and cognitive outcomes were tracked within the same assessment framework. Shen et al. (26) reported comparable co-benefits in their RCT, with cognitive performance improving alongside motor and fitness outcomes.

Hawks et al. (37) approached the cognitive dimension differently: social communication deficits, operationalized as a neurodevelopmental characteristic affecting the capacity to interpret and produce communicative signals during motor learning, rather than as a social participation outcome, negatively predicted motor skill acquisition (*β* = −0.21), while motor coordination positively predicted it (*β* = 0.31), pointing to an interaction between neurocognitive functioning and physical learning that the other studies do not model. This variable was therefore classified under the cognitive dimension rather than the social dimension of PL.

The remaining five studies addressed cognitive constructs incidentally, through implementation intentions as a predictor of PA behavior (41) or food literacy knowledge as a secondary outcome (29), without examining how movement knowledge develops or how SEN children understand and apply motor concepts within PE.

#### 3.3.4 Social dimension

Social outcomes were addressed in five studies. In all five, the social dimension was operationalized as a contextual feature, the presence of peers, rather than as a PL competency to be tracked and developed. No study measured interpersonal skills, cooperative engagement, or social communication as distinct PL outcomes. Chiva-Bartoll et al. (25) reported significant improvements in moderate PA (*p* ≤ 0.001) and manual dexterity and balance (*p* ≤ 0.05) following a service-learning program; qualitative findings indicated enhanced social inclusion and improved parental perceptions of school integration, though no validated social outcome measure was used. Shen et al. (26) incorporated facilitated peer interaction as a deliberate program component and found significant quality-of-life gains (*η*²=0.439), but again without independent measurement of social participation. The observational data from Stanish et al. (42) offers a counterpoint: 32% of adolescents with ASD considered physical activities too hard to learn, and only 68% believed PA was a way to make friends, compared with 97% of typically developing peers (*p* < 0.001), a gap that suggests social PA beliefs in SEN populations differ fundamentally from the normative assumptions embedded in inclusive PE models. No study measured social participation as a distinct, validated PL outcome.

### 3.4 Ecological design and multi-level factors

For this review, a study was coded as ecological when it explicitly engaged with at least two systemic levels beyond the individual (e.g., family, peers, school organization, community, or policy) either as components of the intervention design or as analytically distinct factors in the study. Partial ecological framing was coded when ecological language or frameworks were referenced but not operationalized across levels. Studies focusing exclusively on individual-level outcomes without reference to contextual factors were coded as non-ecological.

Seven of the 22 studies adopted a genuinely ecological approach (26, 27, 32, 37, 40, 41, 43). Five studies demonstrated partial ecological framing (24, 25, 42, 44, 45). The remaining ten studies operated exclusively at the individual level, with no explicit engagement with contextual or systemic factors.

Among studies with ecological framing, the school and institutional level was the most consistently engaged, present in all seven fully ecological studies. Teachers appeared as key implementation agents in school-based programs. Moreover, the quality and intensity of teacher professional development emerged as a critical variable. For example, Noetel et al. (27) demonstrated in their cluster RCT that teacher completion rates significantly predicted students' FMS competency at post-test (*b* = 10.40, 95%CI [1.94, 18.87], *p* = 0.016), while the low-intensity professional learning provided to the broader sample was associated with null intervention effects. Jin and Yun (41) further documented the moderating role of instructional context, lesson content (*β* = 0.56, *p* < 0.001) and classroom location (*β*=−.95, *p* < 0.001) significantly moderated the relationship between teacher PA-promotion behavior and student physical activity, illustrating the structural power of the school environment as an ecological lever.

Family involvement was present as an active program component in four studies. Shen et al. (26)incorporated bi-weekly parent–child sessions and a parent education component within their ICF-based APA program. Healy and Marchand (44) positioned parents as the primary mediators of intervention delivery in Project CHASE, a Facebook-based program that explicitly drew on an ecological approach to leverage natural home and community settings. Curtin et al. (29) required family participation for remote program delivery, with parents preparing materials and co-administering assessments via Zoom.

Community-level engagement was least systematically operationalized. Hawks et al. (37) embedded the iCan Bike camp within a community organization (Missouri Families for Effective Autism Treatment), with parents as co-participants and volunteer spotters providing peer-level support. Stanish and Temple (46), while not coded as fully ecological in design, integrated community infrastructure (YMCA fitness centres) as the intervention setting and recruited typically developing peers as workout partners, creating an interpersonal ecological layer. Policy-level engagement was limited to citations of national or international guidelines (WHO, USDHHS, AAP) in four studies, without operationalizing policy levers as active intervention components.

Finally, the predominance of individual-level studies and the shallow engagement with ecological levels beyond the school represents a significant gap in the current evidence base. No study simultaneously intervened across individual, family, institutional, and community levels with a formally specified ecological model, despite the well-documented relevance of socio-ecological frameworks for promoting physical activity in SEN populations. The absence of multilevel ecological intervention designs limits the field's capacity to identify which contextual factors drive or inhibit PL development, and for whom.

### 3.5 Contextual barriers and facilitators to PL and physical activity promotion

Across the 22 included studies, several contextual factors emerged consistently as facilitators or critical barriers to the promotion of PL and physical activity among children and adolescents with SEN.

#### 3.5.1 Facilitators

Among the facilitators of PL promotion, peer-mediated strategies produced the most consistent positive outcomes across program types, populations, and settings. Indeed, structured peer interaction improved physical outcomes and affective engagement simultaneously (25, 35, 46). In addition, gains were not limited to SEN participants; peer partners themselves showed comparable improvements in fitness outcomes in the only study that measured them (46).

The learning environment also independently influenced outcomes. Programs emphasizing enjoyment, autonomy, and positive reinforcement over performance goals yielded better affective outcomes than those focused only on motor skills, both for individuals with ASD and those with intellectual disabilities (28, 36, 40). The contrast with standard physical education is striking: Gobbi et al. (35) showed that the same students experienced more enjoyment during peer tutoring sessions than during their regular physical education classes, even though the physical content remained unchanged.

Finally, technology amplified engagement under specific conditions. In studies where digital tools were embedded within a supervised, structured school environment, compliance and attendance were substantially higher than when the same tools were used without institutional support (38, 45).

#### 3.5.2 Barriers

Among the barriers to PL promotion, teacher preparation was the primary implementation constraint. Across school-based studies, the quality and dosage of teacher training determined whether intended physical activities reached students effectively (27, 41). Low-intensity professional development was insufficient to change outcomes even in well-designed programs.

Another distinct category of barriers concerns participation itself. Sensory sensitivities, fear of injury, and social stigma reduced willingness to engage in non-specialized settings, particularly among adolescents with ASD (42). These barriers are environmental and perceptual rather than motivational.

### 3.6 The setting gap: genuine inclusion versus segregated practice

Settings were classified according to the four operationalized categories defined in Section 2.4. Four of the 22 studies were conducted in genuinely inclusive settings, where SEN students participated alongside typically developing peers with structured interaction: two school-based (35, 41), one community-based (46), and one single-subject study in an inclusive elementary school (43). Two studies were partially inclusive: one comparing disability and non-disability groups across shared PA contexts without direct structured interaction (24), and one involving pre-service PE teacher students as external partners rather than embedded peers (25). Eight studies were conducted in segregated or specialized settings. The remaining eight did not provide sufficient information to determine the inclusive character of the setting.

Table 4 provides a comprehensive synthesis of the distribution of PL dimensions across SEN populations, settings, and PL frameworks for all 22 included studies.

## 4 Discussion

The present study aimed to map the conceptualization and operationalization of PL in physical activity programs for children and adolescents with disabilities. The 22 included studies show that the literature is still developing and refining its use of the core concept. The findings document a field in which the PL construct is more often assumed than engaged: its motor dimension is measured, its affective, cognitive, and social dimensions are not. Section 3.2 details the evidence; what follows attempts to explain it. For researchers working at the intersection of PL and SEN, this gap between the richness of the theoretical concept and the impoverishment of its empirical operationalization constitutes the central problem that the field must address. The social dimension illustrates this gap most sharply: defined in PL frameworks as the capacity to engage, cooperate, and connect with others through movement (13), it was reduced in the corpus to a contextual condition, the presence of peers, rather than treated as a competency to measure and develop. Research on group-based PA programs for youth with disabilities consistently reports gains in social interaction and peer engagement when this dimension is explicitly targeted (47), suggesting that the tools exist, but have not yet been integrated into PL-focused SEN research.

### 4.1 A familiar gap, sharpened by context

The predominance of the physical dimension in studies involving adolescents with SEN (i.e., present in 86% of studies) is coherent with the literature on PL. Carl et al. (15), in the first meta-analysis of PL interventions across populations, found the same hierarchy: physical competence generated the largest effects (SMD 0.90), while motivation and confidence produced the weakest (SMD 0.30). The specificity of this corpus is the depth of the conceptual absence. Crucially, In Carl et al.'s meta-analysis, all included studies explicitly used PL as a theoretical underpinning or evaluation outcome, which was an inclusion criterion. In the present review, only three of 22 studies explicitly used PL. The remaining 19 produced what might be called implicit or absent PL research: work that addresses physical activity outcomes in SEN populations without situating those outcomes within a PL framework.

This could be explained by the discipline and theoretical frameworks employed. The concept of PL, as a holistic and philosophically grounded construct, remains largely confined to English-speaking research communities (Canadian, British, and Australian) and to a specific branch of physical education. Researchers studying physical activity in the context of students with SEN often come from the fields of adapted physical activity, rehabilitation science, or health promotion, where the dominant frameworks are biomedical or behavioral rather than phenomenological (18, 19). They use the concepts they are familiar with, motor skills tests, fitness test batteries, measures of behavior during physical activity, and produce solid and useful evidence, but this evidence nevertheless leaves the affective, cognitive, and social dimensions of PA unevaluated and therefore invisible. This is the consequence of work at the intersection of two fields that have not yet developed a common conceptual language.

The three studies that explicitly adopted a PL framework (e.g., 28, 29, 32) reported significant changes across multiple PL domains simultaneously, including affective and cognitive outcomes that the rest of the corpus rarely measured. This pattern is consistent with the possibility that SEN children can develop motivation, confidence, and movement knowledge when programs are designed to foster these qualities and when instruments are in place to detect them, though three studies are insufficient to establish this as a generalizable finding. The 19 studies that did not adopt a PL framework did not find affective and cognitive effects. However, they did not look for them either. Therefore, the field may be systematically underestimating what SEN children are capable of, simply because the dominant assessment tools are not designed to capture it. Building and validating multidomain PL instruments for SEN populations is therefore not only a methodological priority, but a critical step toward generating the evidence needed to assess what SEN children are capable of when holistic frameworks are applied.

### 4.2 The setting gap: a research culture problem

Only four of 22 studies were conducted in genuinely inclusive settings. Conducting rigorous intervention research in mainstream inclusive schools is structurally more difficult than working in specialized settings: access requires the cooperation of general physical education teachers and school administrations; consent procedures involve the families of typically developing students alongside those of SEN students, and the heterogeneity of mainstream classes complicates both intervention delivery and outcome measurement. These elements are real obstacles. Addressing this will require sustained partnerships between researchers, general PE teachers, and adapted PE specialists, partnerships that take time to build but that are a prerequisite for producing evidence relevant to mainstream inclusive practice.

That said, the consequences for the evidence base are serious. What is currently known about PL development in SEN populations is almost entirely derived from environments that do not reflect where most SEN children spend their school days. Inclusive education is the legislative and ethical norm across the countries represented in this corpus, yet the research that is supposed to inform inclusive physical education practice is built on evidence from segregated contexts. This disconnect suggests that practitioners in mainstream schools have almost no empirically grounded guidance for how to develop PL in heterogeneous classes that include SEN students alongside their typically developing peers.

The four genuinely inclusive studies hint at what such guidance might look like. Across contexts as different as Korean middle-school inclusive PE (41), Italian secondary schools (35), a YMCA community program in the United States (46), and an inclusive elementary school in Indonesia (43), the consistent element was structured peer interaction, not undifferentiated co-presence, but deliberate pairing with role and purpose. Peer interaction and cooperative learning can be, and are, mobilized in specialized settings as well. What inclusive settings add is the presence of typically developing peers a condition that, based on the present corpus, appears to expand the social and relational dimensions of physical activity (35, 46), though whether this translates into differential PL outcomes compared to well-designed cooperative approaches in specialized settings remains to be empirically established. Whether this mechanism is generalizable beyond these small, non-randomized studies is a question the current evidence cannot answer.

### 4.3 What the ecological data reveal about implementation

Analyzing the barriers and facilitators data using an ecological approach reveals that the factors determining whether PL programs succeed are multidimensional and multilevel.

The individual-level data challenge a common assumption that low PA participation in SEN students primarily reflects their impairment. Bentzen and Malmquist (24) showed that the gap in psychological need satisfaction between students with and without disabilities disappeared in self-organized physical activity, appearing only in mandatory PE and organized sport. Fear of injury, sensory discomfort, and the perception that activities are too hard to learn, documented in Stanish et al. (42), are not fixed characteristics of ASD, but rather responses to environments that have not been designed with these students in mind. This interpretation is consistent with the broader qualitative literature. A recent meta-synthesis of 13 studies found that environmental design, peer attitudes, and teacher competence rather than disability *per se* were the primary determinants of PA participation in integrated school settings (5). The individual-level barriers, in other words, are at least partly associated with the institutional conditions in which students are placed.

At the interpersonal level, peer interaction emerged as a consistently effective lever. It appeared to be a mechanism that shifted physical and affective outcomes simultaneously. Programs pairing SEN students with typically developing peers consistently reported improvements across both dimensions, and in the one study that measured peer partners directly (46), typically developing participants showed comparable fitness gains. This finding may encourage teachers who resist inclusive adaptations out of concern for typically developing students: peer-mediated formats appear to benefit both groups. Family involvement, where operationalized as an active program component rather than passive consent, also shifted outcomes, particularly in remote contexts where parents became co-facilitators (29, 44).

At the institutional level, the results suggest that teacher preparation, not program content, was consistently associated with whether interventions reached their intended outcomes. The null result in Noetel et al. (27) makes this point forcefully: teacher completion rates, not the program content, predicted student outcomes. Jin and Yun (41) reached a similar conclusion from observational data: lesson content and classroom location moderated the relationship between teacher PA-promotion behavior and student physical activity far more than direct instruction did. Read together, these two studies, conducted a decade apart, in South Korea and Australia respectively, with different populations and designs, point to the same structural constraint. A well-designed program delivered by an undertrained or under-supported teacher is, on this evidence, unlikely to produce durable change. This aligns with Carl et al.'s (15) call for a tighter interlocking between PL theory and intervention delivery at all stages, and with evidence that sustained teacher professional development can durably change both pedagogical practice and PL operationalization in the classroom (16), suggesting a practical reorientation of priority from program innovation toward teacher development.

At the environmental and policy level, no study in this corpus simultaneously intervened across individual, interpersonal, institutional, and community levels within a formally specified ecological model (6). Technology appeared as a facilitator only when embedded within environments where the other conditions were already in place: supervised VR exergaming produced engagement, while the same headsets used at home without supervision produced disengagement (45). Wearable trackers achieved unusually high compliance in ASD students, attributed to preferences for routine and visual feedback, but only within a structured school environment with teacher support (38). The available evidence, though limited to a small number of studies, suggests that technology amplifies existing conditions rather than substituting for them. A policy agenda that treats digital tools as a primary solution to structural inclusion problems may therefore be misplaced.

### 4.4 Implications for practice and policy

The data examined here highlight several concrete avenues for action. For example, physical education teachers can influence the learning climate. Across the included studies, programs that prioritized enjoyment, autonomy, and positive reinforcement over normative performance consistently reported stronger affective outcomes, regardless of the specific physical content delivered. Instructional climate adjustments of this kind do not require structural changes or additional resources, suggesting they represent a feasible starting point for teachers working within existing constraints. The results of Salaun et al. (36) are particularly instructive: a strengths-based program improved physical self-perception and reduced body dissatisfaction in obese adolescents with intellectual disabilities, without negatively impacting self-esteem. This demonstrates that the way physical education is designed is as important as its content.

Teacher trainers should be able to foster continuous professional development, incorporating personalized support, such as that assessed by Edwards et al. (16) in primary schools. This has direct implications for initial teacher training, where inclusive physical education and adapted physical activity are often considered optional specializations rather than fundamental skills. More broadly, the integration of PL principles into professional training programs, whether in adapted physical activity, healthcare, or teacher education, remains an under addressed priority. As Roetert and Jefferies (48) argued, embracing PL as a conceptual framework across the curricula and certifications of practitioners in education, sport, and health sectors is a prerequisite for the kind of coherent, cross-sector approach that SEN populations require. The present review suggests that this integration has not yet occurred: most of the researchers who study physical activity in SEN contexts come from fields where PL is not part of the professional vocabulary, which is itself part of the explanation for the conceptual fragmentation documented here.

The assessment infrastructure gap deserves attention from adapted physical activity specialists and SEN associations. The absence of validated, multidimensional PL tools adapted for SEN populations is not a peripheral methodological concern. It is what makes the affective, cognitive, and social dimensions of PL invisible in research and practice alike (18, 19). Investing in the systematic adaptation of instruments like the CAPL-2 for diverse SEN profiles, through cognitive interviewing with SEN youth, caregiver and teacher proxy measures where self-report is not feasible, and differential item functioning analyses to assess measurement equivalence across diagnostic groups, would enable both researchers and practitioners to measure what they are trying to develop. The rare studies that have attempted multidomain PL operationalization in SEN contexts demonstrate that this is feasible (28, 29).

Finally, the structural disconnect identified in this review, between inclusive education mandates and a research base built almost entirely on segregated settings, is a funding and policy problem as much as research one. Children and adolescents with SEN remain disproportionately inactive and underserved by the existing evidence base (4), in part because the research conditions that would generate relevant evidence, access to mainstream schools and partnerships between general and adapted PE specialists, are not systematically supported (5, 17). Mechanisms that create genuine access for researchers to mainstream inclusive schools, and that incentivize such partnerships, would substantially improve the conditions for generating the evidence that inclusive education policy currently requires.

## 5 Perspectives and limitations

The studies reviewed here show that PL outcomes can be improved in SEN children through well-designed programs, but the conditions under which this happens are narrow, the evidence base thin, and the settings largely disconnected from mainstream inclusive education. The field has not yet produced the type of evidence needed to guide policy and practice at scale: studies are generally small, designs are predominantly quasi-experimental, follow-up periods are short, and many interventions operate in segregated or specialized settings rather than in the inclusive school environme*n*ts increasingly required by disability policy.

Future studies should prioritize genuinely inclusive school settings as the primary context, not as an aspirational ideal but as a methodological requirement. Interventions should be embedded within mainstream PE curricula, engaging typically developing peers as active participants rather than passive bystanders, and evaluated using multilevel, ecologically valid frameworks capable of capturing the physical, affective, cognitive, and social dimensions of PL simultaneously. Teachers trained to support heterogeneous groups constitute a minimum condition for generating evidence relevant to the actual school experience of many SEN children. Robust longitudinal designs with adequate follow-up are needed to establish whether early PL gains are sustained over time and whether they translate into lifelong physical activity participation.

Future research should adopt an explicitly ecological and multilevel perspective, treating the school not as a neutral delivery platform but as a complex social system in which individual, interpersonal, institutional, environmental, and policy factors interact to shape physical activity behavior (6, 24, 41). Interventions acting on a single level are unlikely to produce durable change in a system where contextual moderators routinely outweigh program content. Such approaches must also attend to the heterogeneity of SEN: the barriers and facilitators identified in this review differ across ASD, intellectual disability, and mixed SEN profiles, suggesting that a single model is unlikely to be adequate across all.

Finally, the methodological infrastructure of the field needs to mature. The proliferation of heterogeneous assessment tools, with no single instrument capable of capturing the full multidimensional scope of PL in SEN populations, limits cumulative knowledge building. The development and validation of PL assessment tools specifically designed for SEN populations, drawing on established frameworks for instrument adaptation and content validity, would substantially increase the comparability of future research. These directions point toward a new generation of studies that is larger in scale, longer in duration, more ecologically grounded, and more explicitly anchored in the theoretical commitments of PL. Fundamentally, the PL approach is based on the principle that all bodies, regardless of their abilities, have the capacity to experience movement as enriching, and the right to receive instruction that respects this capacity (16, 17). The field has not yet established the necessary evidence base to make this principle applicable to children with SEN in inclusive settings. This is the work that lies ahead.

This review is subject to several limitations that should be considered when interpreting its findings. First, despite searching four electronic databases, the possibility of relevant studies being missed cannot be excluded, particularly for grey literature and non-English language publications, a language bias that Carl et al. (15) also acknowledged in the broader PL intervention literature. Furthermore, the search strategy was intentionally anchored in the most widely indexed PL-adjacent terms. Items specific to cognitive PL dimensions (e.g., movement knowledge, implementation intentions) and social PL dimensions (e.g., social participation through movement, cooperation) were not included as primary search terms, as these constructs are inconsistently indexed across the searched databases. This choice may have underrepresented studies addressing these dimensions as primary outcomes and should be considered when interpreting the finding that cognitive and social dimensions are systematically underrepresented in the corpus.

Second, the findings of the review should be interpreted in the context of a potential publication bias (49, 50). Publication bias is the tendency of researchers and journal editors not to publish studies that fail to produce significant effects. This paper is based only on published studies, and this limitation must be considered when interpreting our results.

Third, citation chasing (backward and forward) was not conducted systematically, which may have resulted in relevant studies being missed, particularly older foundational work and very recent publications not yet indexed in the searched databases.

Fourth, no formal quality appraisal was conducted, consistent with scoping review methodology but limiting the extent to which findings can be weighted by study rigor.

Fifth, the small sample sizes and predominantly quasi-experimental designs of the included studies mean that effect size estimates, where reported, should be interpreted with caution and are not suitable for meta-analytic synthesis.

These limitations notwithstanding, this review provides the most comprehensive map to date of how physical literacy is conceptualized and operationalized for SEN children and adolescents in educational and community physical activity contexts.

## 6 Conclusion

This scoping review mapped how PL is conceptualized and operationalized in educational and community-based physical activity contexts for children and adolescents with special educational needs, drawing on 22 studies published between 2012 and 2025. Three main conclusions emerged.

First, the explicit approach to the concept of physical literacy remains limited. Only three studies explicitly anchored their work within a recognized framework of PL. Most of them have reduced PL to its motor dimension, while largely neglecting the affective, cognitive, and social pillars. This trend reflects the conceptual fragmentation documented in the broader literature on PL (15), but appears more pronounced in the context of SEN.

Second, the available data are structurally disconnected from inclusive school settings. Only four of the 22 studies were conducted in genuinely inclusive environments, highlighting an important gap in the literature. In these studies, peer-interaction-based formats consistently yielded positive outcomes, both physically and socially. Current data do not allow us to determine whether social co-participation is a necessary condition for the development of PL in children with special educational needs, or whether equivalent results can be achieved through well-designed cooperative approaches in specialized settings.

Third, contextual factors (e.g., teacher professional development, school climate, peer interaction structures, and family involvement) have repeatedly moderated program effectiveness across all ecological levels. Yet, most interventions addressed only one or two of these factors simultaneously, thereby limiting their scope and potential sustainability.

These findings highlight the need for a clear and urgent plan of action. The field calls for larger-scale, longitudinal, and multi-level studies conducted in truly inclusive school settings, evaluated using validated PL measurement tools designed for and with students with SEN, and grounded in ongoing teacher training rather than solely in program innovation.
